# Abnormal Plasma/Serum Magnesium, Copper, and Zinc Concentrations Associate with the Future Development of Cardiovascular Diseases

**DOI:** 10.3390/nu17091447

**Published:** 2025-04-25

**Authors:** Boyang Lin, Robin Alexander, Remi Fritzen, Sarah Mills, Alan J. Stewart, Colin McCowan

**Affiliations:** School of Medicine, University of St. Andrews, St. Andrews KY16 9TF, UK; bl51@st-andrews.ac.uk (B.L.); rnaa1@st-andrews.ac.uk (R.A.); rf79@st-andrews.ac.uk (R.F.); seem1@st-andrews.ac.uk (S.M.); cm434@st-andrews.ac.uk (C.M.)

**Keywords:** plasma magnesium, plasma copper, plasma zinc, major adverse cardiovascular events, health records

## Abstract

**Background/Objectives**: Cardiovascular diseases (CVDs) are the leading cause of global mortality. Major adverse cardiovascular events (MACEs)—such as acute myocardial infarction, stroke, and heart failure—are critical endpoints in the clinical research. The existing research has shown metal ions are important regulators of cardiovascular functioning, and defective metal handling may be associated with an increased risk of CVD. This study examines the association of the plasma/serum levels of magnesium, copper, and zinc with MACE incidence and the prevalence of circulatory system diseases, by using electronic health records from a subset of the Scottish population. **Methods**: We categorised individuals by high, low, or normal plasma/serum metal levels, and calculated the percentage of those who subsequently developed a MACE, identified using related International Classification of Diseases, 10th Revision codes from hospital admission records. Logistic regression was employed to analyse the association between pre-event metal ion levels and the development of specific circulatory system disease subgroups. **Results**: This study found abnormal magnesium, high copper, and low zinc were associated with a higher risk of developing MACEs. Low magnesium, high copper, or low zinc were associated with increased risks of various circulatory diseases, with specific variations, like low copper increasing venous and lymphatic disease risk. **Conclusions**: Our findings suggest abnormal plasma metal profiles are associated with the development of MACEs and circulatory disease events, underscoring the importance of monitoring plasma metal levels for cardiovascular risk management and prevention.

## 1. Introduction

Cardiovascular diseases (CVDs) are the predominant cause of global mortality. Based on the data from the World Health Organisation (WHO), around 17.9 million people globally died from CVDs in 2019, which contributes to 32% of all global deaths [1]. In the Lancet’s Global Burden of Disease in 2021 [2], cardiovascular diseases (heart disease and stroke) represent two of the top-five global disease causes of death. Metal ions are crucial for numerous processes linked to both cardiac and vascular functioning, and disrupted metal ion homeostasis thus has the potential to contribute to the development of CVD [3]. For example, Mg^2+^ is vital in regulating cardiovascular functioning and the maintenance of blood pressure through the control of cellular membrane potential; it helps to activate Na^+^, K^+^-ATPase and counteracts some of the effects of Ca^2+^ [4,5]. Mg^2+^ is known to have anti-inflammatory and antioxidant properties, which may improve lipid metabolism and reduce atherosclerosis risk [5,6]. Copper (Cu^+/2+^ ions) serves as a cofactor for many enzymes, facilitating oxidation-reduction reactions [7]. Adequate copper levels are vital for cardiac mitochondrial function and energy production, supporting normal cardiac functioning [8]. However, excess copper has been associated with oxidative stress, impaired lipid metabolism, and cell death, contributing to the progression of CVD [9]. Similarly, Zn^2+^ plays an essential role in cardiovascular health through its influence on haemostasis, oxidative stress, inflammation, and Ca^2+^ homeostasis, with its transporters playing key roles in the maintenance of cellular Zn^2+^ balance, impacting on heart function and vascular integrity [10,11,12]. An adequate balance of these key nutrients over the life course is thus essential for minimising CVD risk.

In clinical research, the incidence of major adverse cardiovascular events (MACEs) has increasingly become a critical primary endpoint [13,14,15]. This study aims to examine potential associations between abnormal plasma levels of zinc, copper, and magnesium and future MACEs, including examining specific subgroups of circulatory system diseases. We interrogated electronic health records (EHRs) from the National Health Service (NHS) Tayside and Fife Health Boards in Scotland, UK, utilising a comprehensive set of International Classification of Diseases, 10th Revision (ICD-10) codes to identify future MACEs in our patient cohort. Through this approach, we aim to provide a more precise assessment of CVD risk and offer insights for targeted public health and clinical interventions.

## 2. Materials and Methods

### 2.1. Data Source

This study utilised EHRs from the NHS, covering the Tayside and Fife regions in Scotland, which together encompass approximately 20% of the Scottish population [16]. The data were hosted by The Health Informatics Centre (HIC) at the University of Dundee, Dundee, UK and were provided in an anonymised form. The datasets were accessed within a Safe Haven environment and processed using R studio (2022.02.1). All data exported from Safe Haven were reviewed and approved for disclosure control.

The datasets employed in this research encompassed a variety of clinical records, including Scottish Morbidity Records (SMRs) from “Outpatients” (SMR00), “Hospital Admissions” (SMR01), “Psychiatric” (SMR04), “Cancer Register” (SMR06), and “Accident and Emergency” (AE). Additionally, this study utilised the “Biochemistry laboratory” dataset and the “Demography” record. These datasets were extracted in 2022, covered the years 2000 to 2021, and were linked through the Community Health Index (CHI) numbers, a unique identifier for every patient registered with a general practitioner in Scotland. To ensure privacy, the CHI was transformed into a project-specific pseudo-identifier.

### 2.2. Overall Populations and MACE Patient Identification

The study population comprised individuals aged between 18 to 100 years, divided into the following age groups: 18–19, 20–29, 30–39, 40–49, 50–59, 60–69, 70–79, 80–89, and 90+ years. Patient demographics were studied to identify the numbers of male and female participants. To identify patients who experienced a MACE, a group of ICD-10 codes, “I00–I78”, “G45”, “G451–G454”, “G458”, “G459”, and “G460–G468”, were used [17], as shown in Table 1. An appearance of any of these codes in a patient’s linked records categorised them as a MACE patient. The time of each patient’s initial MACE was also recorded using the date from the relevant record.

### 2.3. Analysis of Plasma/Serum Magnesium, Copper, and Zinc Concentration Data in MACE Patients

The plasma/serum concentrations of zinc, copper, and magnesium from test records were extracted from the “Biochemistry” dataset. Only laboratory records from patients recorded prior to their first documented MACE were selected. For each MACE patient, the most recent pre-MACE test records were identified for each test of interest. For the control group, which consisted of individuals with biochemical test records but without MACEs, only the most recent test records were considered.

The concentration of the given metal in each biochemical test result was classified as “high”, “normal”, or “low” based on the reference intervals provided in the records. In cases where reference intervals were not available, the most-reported high and low thresholds in the dataset were used. For copper, the most-reported high threshold was >22 µmol/L, and the low threshold was <10 µmol/L. For zinc, the high threshold was >18 µmol/L, and the low threshold was <10 µmol/L. For magnesium, the most-reported high threshold is >1 mmol/L, and the low threshold was <0.7 mmol/L.

The distribution of control and MACE patients within the “high”, “normal”, and “low” categories for each biochemical test was quantified. The chi-squared test was used to assess significance by comparing the percentages of MACE patients across different metal-status categories (high or low, against the normal levels). Note that all *p*-values are presented at nominal values.

### 2.4. Analysis of Plasma/Serum Magnesium, Copper, and Zinc Concentration Data in MACE Patients

The ICD–10 codes “I00–I02”, “I05–I09”, “I10–I15”, “I20–I25”, “I26–I28”, “I30–I52”, “I60–I69”, “I70–I79”, “I80–I89”, and “I95–I99” were used to classify patients into specific subgroups of circulatory system diseases within the dataset. For each code group, a separate patient list was generated, including the onset date for each disease subgroup. Individuals with magnesium, copper, and zinc test records were categorised as either patients or controls based on their inclusion in the respective patient list. For patients, only test records obtained prior to disease onset were included in the analysis.

Plasma or serum metal concentrations in the records were classified as “high”, “normal”, or “low”, using the method described in Section 2.3. A logistic regression model was employed to evaluate the relationship between the dependent variable (patient’s status: patient vs. control) and the independent variable (magnesium, copper, and zinc status). The reference level for patients’ status was set to “control”, and the reference level for magnesium, copper, and zinc status was set to “normal.” The model was fitted using the generalised linear model (glm) function in R studio (2022.02.1), with a binomial family and a logit link function. Odds ratios (ORs) were calculated by exponentiating the model coefficients (exp_coef_). The 95% confidence intervals (CIs) for the OR were calculated using Equations (1) and (2):

CI_low_ = exp (E − 1.96 × SE) (1)

CI_upper_ = exp (E + 1.96 × SE) (2)

In each equation, E is the estimated coefficient and SE is its standard error.

## 3. Results

### 3.1. Magnesium, Copper, and Zinc Status in MACE Patients and Control Groups

**Figure 1:**
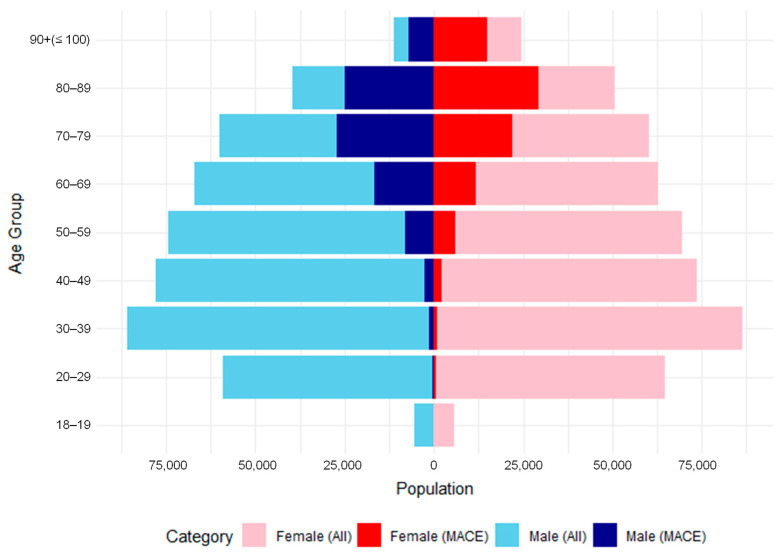
MACE patient demographics, including age and sex. The plot was generated using the ggplot2 package in RStudio (version 2023.06).

Firstly, the overall population was characterised, and individuals who experienced MACEs were identified in the dataset. As summarised in Figure 1, the dataset includes 978,759 individuals aged between 18 and 100 years, of whom 176,350 experienced a MACE. Specifically, among the 498,136 females, 17.6% (87,720) developed a MACE, while 18.4% (88,630) of the 480,623 males were affected.

**Figure 2:**
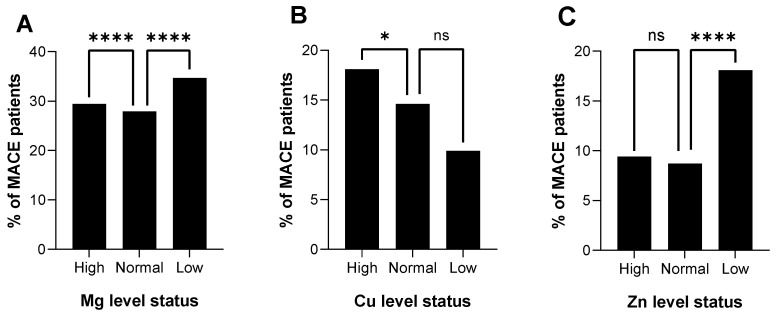
Distribution of MACE patients in high, normal, and low groups for (**A**) magnesium, (**B**) copper, and (**C**) zinc data. Statistical significance was assessed using the chi-squared test. For MACE patients, the most recent plasma test results prior to the event were used, while for the control group, the most recent available test records were employed. Detailed numerical data are presented in Table 2. **** *p* < 0.001, * *p* < 0.05, and ns denotes “not significant”.

To assess the association between metal levels and the risk of developing a MACE, the proportion of MACE patients within each metal concentration group was calculated. This analysis was based on the most recent pre-MACE metal measurements for MACE patients and the latest available metal-level records for the control group. Figure 2 illustrates the distribution of control and MACE patient groups across different magnesium, copper, and zinc status levels (high, normal, and low).

For magnesium, the proportion of MACE patients was 29.4% in the high group and 27.9% in the normal group, rising to 34.7% in the low group. The chi-square test indicated statistically significant differences in the prevalence of MACE patients when comparing high versus normal magnesium levels, with a *p*-value < 0.0001. Similarly, the comparison of low vs. normal magnesium levels revealed a *p*-value < 0.0001, showing elevated MACE rates in both low- and high-magnesium groups relative to the normal range (Table 2). These findings highlight a significant association between magnesium levels and the risk of MACEs, underscoring the potential health implications of abnormal plasma magnesium concentration. Regarding copper status, MACE patients were most prevalent in the high group (18.1%), followed by the normal (14.6%) and low (9.9%) groups. The chi-squared test indicated a significant *p*-value of 0.0316 when comparing the high group against the normal group, suggesting that elevated copper levels may be associated with an increased risk of MACEs. For zinc status, the proportion of MACE patients was 9.4% in the high group and 8.7% in the normal group. However, this percentage was 18.1% in the low group. The chi-squared test comparing numbers of MACE patients in low and normal groups yielded a *p*-value of <0.0001, suggesting a statistically significant association between low zinc levels and an increased risk of MACEs.

### 3.2. Logistic Regression Analysis of Magnesium, Copper, and Zinc Status Prior to the Development of Circulatory System Diseases

Logistic regression models were employed to further investigate the association between plasma metal levels and the development of individual circulatory system diseases. These models were designed to analyse how pre-onset levels of magnesium, copper, and zinc in the circulation correlated with the development of each specific subgroup of circulatory system disease.

**Figure 3:**
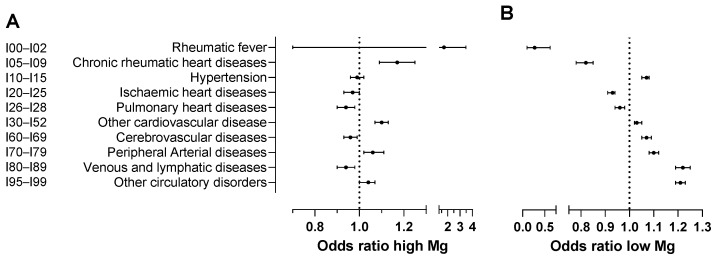
Effects of (**A**) high and (**B**) low magnesium levels on disease occurrence relative to normal levels. Plain regression models were used to analyse the test records for patients and controls. For patients, magnesium test records prior to disease onset were used. The names of the disease classifications are provided in an abbreviated form but correspond to those (based on ICD-10 code) presented in Table 3, Table 4 and Table 5. Error bars in (**A**,**B**) represent 95% confidence intervals.

The relationship between pre-onset magnesium levels and the development of circulatory diseases presented complex patterns (Table 3; Figure 3). In cases of chronic rheumatic heart diseases (I05–I09), high magnesium levels were associated with an increased risk (OR = 1.17, 95% CI: 1.09 to 1.26), whereas low magnesium levels corresponded with reduced risk (OR = 0.82, 95% CI: 0.78 to 0.85), both findings being statistically significant. In contrast, for cerebrovascular diseases (I60–I69), higher pre-onset magnesium levels were linked to a reduced risk (OR = 0.96, 95% CI: 0.93 to 0.99), while lower levels indicated an increased risk (OR = 1.07, 95% CI: 1.05 to 1.09), again both statistically significant.

In ischaemic (I20–I25) and pulmonary (I26–I28) heart diseases, both high and low magnesium levels were associated with decreased risk, with odds ratios of 0.97 (95% CI: 0.93–1.00) and 0.93 (95% CI: 0.91–0.94) for high and low levels in ischaemic diseases, respectively, and 0.94 (95% CI: 0.90–0.98) and 0.96 (95% CI: 0.94–0.98) for pulmonary diseases, respectively. Conversely, in peripheral arterial diseases (I70–I79) and other unspecified disorders of the circulatory system (I95–I99), increased risks were observed with odds ratios of 1.06 (95% CI: 1.02–1.11) for high magnesium levels and 1.10 (95% CI: 1.08–1.12) for low magnesium levels in peripheral arterial diseases, and 1.04 (95% CI: 1.00–1.07) for high magnesium levels and 1.21 (95% CI: 1.19–1.23) for low magnesium levels in other cardiovascular conditions. Additionally, a low magnesium level was associated with a significantly reduced risk of acute rheumatic fever (I00–I02) with an OR of 0.27 (95% CI: 0.11–0.68). In contrast, low magnesium led to a significant increase in the risk of hypertension (I10–I15) and venous and lymphatic diseases (I80–I89) with ORs of 1.07 (95% CI: 1.05–1.08) and 1.22 (95% CI: 1.19–1.25), respectively.

**Figure 4:**
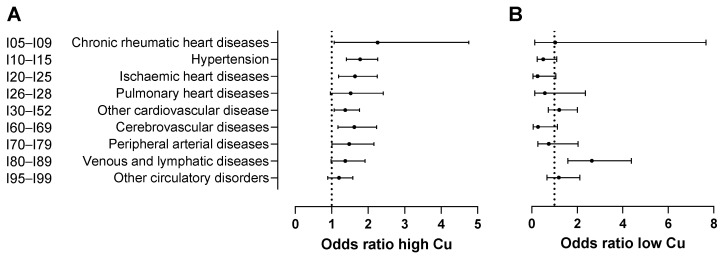
Effects of (**A**) high and (**B**) low copper levels on disease occurrence relative to normal levels. Plain regression models were used to analyse the test records for patients and controls. For patients, copper test records prior to disease onset were used. The names of the disease classifications are provided in an abbreviated form but correspond to those (based on ICD-10 code) presented in Table 3, Table 4 and Table 5. Error bars in (**A**,**B**) represent 95% confidence intervals.

Elevated copper levels were associated with an increased risk of several circulatory conditions (Table 4; Figure 4). Specifically, the OR indicated a more than two-fold risk of developing chronic rheumatic diseases (I05–I09, OR = 2.26, 95% CI: 1.07–4.76) and a significant association with hypertension (I10–I15, OR = 1.78, 95% CI: 1.4–2.26), ischaemic heart diseases (I20–I25, OR = 1.64, 95% CI: 1.19–2.25), other forms of heart disease (I30–I52, OR = 1.37, 95% CI: 1.07–1.76), cerebrovascular diseases (I60–I69, OR = 1.62, 95% CI: 1.17–2.23), and peripheral arterial diseases (I70–I79, OR = 1.48, 95% CI: 1.01–2.16). In contrast, a low copper status significantly heightened the risk for venous and lymphatic diseases (I80–I89, OR = 2.64, 95% CI: 1.59–4.38), as reflected by the reported OR values.

**Figure 5:**
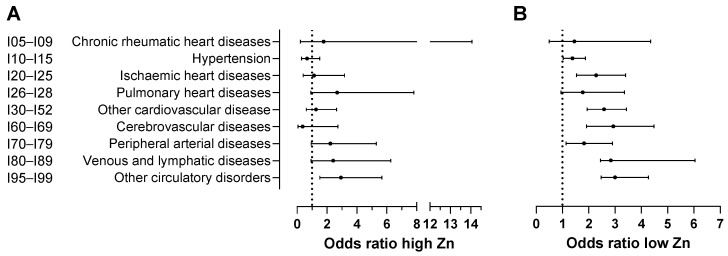
Effects of (**A**) high and (**B**) low zinc levels on disease occurrence relative to normal levels. Plain regression models were used to analyse the test records for patients and controls. For patients, zinc test records prior to disease onset were used. The names of the disease classifications are provided in an abbreviated form but correspond to those (based on ICD-10 code) presented in Table 3, Table 4 and Table 5. Error bars in (**A**,**B**) represent 95% confidence intervals.

A high zinc level prior to disease onset was only found to be significantly and positively associated with the incidence of “other unspecified disorders of the circulatory system” (I95–I99, OR = 2.93, 95%CI: 1.52–5.67; Table 5; Figure 5), while a low zinc level was linked to the progression of a broader range of conditions, including hypertensive diseases (I10–I15, OR = 1.38, 95%CI: 1.02–1.87), ischaemic heart diseases (I20–I25, OR = 2.28, 95% CI: 1.53–3.40), other forms of heart disease (I30–I52, OR = 2.58, 95%CI: 1.94–3.43), cerebrovascular disease (I60–I69, OR = 2.93, 95%CI: 1.91–4.48), peripheral arterial disease (I70–I79, OR = 1.82, 95%CI: 2.44–6.04), venous and lymphatic disease (I80–I89, OR = 3.84, 95%CI = 2.44–6.04), and other unspecified disorders of the circulatory system (I95–I99, OR = 3.00, 95% CI: 2.11–4.27).

## 4. Discussion

This study provides a detailed analysis of the associations between plasma levels of magnesium, copper, and zinc and the occurrence of MACEs and circulatory system diseases using the EHRs of a well-defined Scottish population. Both high and low magnesium levels are associated with an increased risk of developing MACEs, compared to normal levels. Although it is important to note that the difference between high and normal magnesium levels was statistically significant, it was relatively modest (cf. 29.4% vs. 27.9%). Similarly, individuals with high copper and low zinc levels had a higher risk of developing MACEs. Additionally, we examined specific subtypes of circulatory diseases (I00–I99), allowing for a more nuanced understanding of disease-specific risks. Specifically, for circulatory system diseases, our findings suggest that low magnesium, high copper, and low zinc prior to the disease onset are generally associated with an increased risk across most conditions, with specific variations, like low copper, increasing venous and lymphatic disease risk. The use of standardised plasma/serum measurements enhances data reliability, and the findings have potential implications for early disease risk stratification and public health interventions. Nevertheless, our study has limitations, such as relying solely on the latest recorded metal measurement before disease onset for the MACE study. Moreover, all *p*-values are presented at the nominal values and no adjustments for multiple testing were made. Additionally, in the logistic regression model analysis, the unadjusted models may be influenced by unknown confounders, such as sex, age, and socioeconomic status. Although individual dietary habits were not recorded, the population studied is generally representative of a Western dietary pattern [19]. However, due to regulatory mechanisms of absorption and excretion [20], dietary intake does not always directly reflect plasma concentrations, which are more reliable indicators of physiological status [21]. In addition, it is known that diet strongly influences gut microbiota [22], which in turn can influence metal homeostasis in the body, including the metals examined in this study [23,24,25]. This is interesting as the microbiome is a potential target for nutraceutical agents, which may lead to improvements in metal handling [26].

Our findings reveal a complex relationship between magnesium levels and cardiovascular conditions, demonstrating that low magnesium generally correlates with increased disease risk. This aligns with the existing research, which suggests that magnesium deficiency may contribute to vascular stiffness, oxidative stress, and arrhythmias, thereby increasing cardiovascular disease risk. For instance, a cross-sectional study by Tan et al., involving 9708 participants, found that higher magnesium depletion scores were associated with an elevated risk of hypertension [27]. Additionally, a systematic review and meta-analysis reported that lower dietary magnesium intake is linked to an increased stroke risk, underscoring the critical role of adequate magnesium consumption in CVD prevention [28].

Interestingly, our results also highlight a protective effect of low magnesium levels in specific conditions, such as ischaemic and pulmonary heart diseases, with both low and high magnesium levels associated with a reduced risk in these diseases. Furthermore, high magnesium levels were protective against cerebrovascular diseases (I60–I69). Given that only 1% of the body’s magnesium is present in plasma [4], moderate deficiency may trigger compensatory responses, such as enhanced magnesium retention in tissues and bones [29], potentially explaining the observed protective effects at lower levels. Conversely, high levels of magnesium might exert protective effects through vasodilatory, anti-inflammatory, and neuroprotective properties [30,31,32], which could be particularly relevant in ischaemic and cerebrovascular conditions. These findings highlight the nuanced impact of magnesium on cardiovascular health and suggest that both deficiency and excess might influence disease risk differently across specific conditions. Further research is needed to explore whether targeted magnesium interventions can provide clinical benefits.

From our results, high copper levels are associated with increased risks of MACEs and multiple circulatory conditions. These are consistent with the existing studies, which suggest that excessive plasma copper levels may contribute to cardiovascular pathologies through mechanisms such as increased oxidative stress, lipid peroxidation, and inflammatory responses [9]. Furthermore, the epidemiological evidence suggests that elevated circulating copper levels are significantly associated with an increased risk of stroke, coronary artery disease mortality, cardiovascular mortality, and all-cause mortality, with most associations showing moderate certainty of evidence [33].

The findings also suggest that low copper levels, rather than high copper levels, are significantly associated with an increased risk of developing venous and lymphatic diseases (I80–I89). Copper plays a crucial role in maintaining the structural integrity of the heart and blood vessel walls by supporting the activity of the copper-dependent enzyme lysine oxidase, which facilitates the cross-linking of elastin and collagen [34]. Consequently, copper deficiency may lead to weakened vessel walls and subsequently to vascular dysfunction. Beyond its structural role, copper is also essential for both innate and adaptive immunity. Copper deficiency alters immune cell activity and composition and disrupts the production and secretion of immunoreactive substances. This includes a reduction in neutrophil count and the downregulation of cytokine IL-2 secretion by T cells [35]. Additionally, copper deficiency impairs lymphatic cholesterol transport and peripheral lipolysis of chylomicrons in rats, potentially leading to lymphatic dysfunction and congestion [36]. These disruptions can further contribute to lipid accumulation, vascular inflammation, and endothelial dysfunction [37]. Together, these mechanisms likely underlie the increased risk of venous and lymphatic diseases observed in individuals with low copper levels, highlighting the importance of adequate copper intake for vascular and immune health.

Consistent with the previous studies, our findings demonstrate that low zinc levels are associated with increased risks of MACEs and multiple cardiovascular conditions. Zinc plays a crucial role in regulating vascular tone, endothelial function, oxidative stress, blood pressure, and lipid metabolism. A deficiency in zinc has been linked to endothelial dysfunction, increased inflammation, arterial stiffness, atherosclerosis, and thrombotic risk [10,38,39]. These mechanisms provide a plausible explanation for the broad association between low zinc levels and various cardiovascular conditions. A meta-analysis of 27 case–control studies found that low serum zinc levels were associated with a higher risk of heart failure, suggesting that maintaining adequate zinc levels may be cardioprotective [40]. Another meta-analysis of 13 studies (2886 subjects) found that patients with myocardial infarction (MI) had significantly lower zinc levels in serum and hair compared to healthy controls. This association was stronger in Asian populations than European populations, and both men and women with MI had lower zinc levels [41].

Notably, our results suggest that a high zinc level is associated with an increased risk of other cardiovascular diseases (I95–I99). Some evidence suggests that excessive zinc intake may disrupt copper metabolism [42] and alter oxidative stress pathways, leading to reduced nitric oxide (NO) bioavailability [43], which in turn may contribute to vascular dysfunction. To our knowledge, no direct evidence currently establishes a causal relationship between zinc levels and specific diseases within the other and unspecified disorders of the circulatory system (I95–I99) category. Hypotension remains an underexplored area in the zinc research. One study reported a negative correlation between serum zinc and diastolic blood pressure [44], suggesting a potential role of zinc in blood pressure regulation. However, as this study primarily focused on hypertension, the effects of zinc on hypotension remain largely unknown. Future research should aim to elucidate the role of zinc in influencing vascular tone and circulatory homeostasis.

The potential clinical applications of our findings include considering plasma magnesium, copper, and zinc levels as potential risk factors for CVD, facilitating early screening and target monitoring for high-risk individuals. It is possible that both high and low plasma magnesium levels are protective, although a causative association between these and CVD risk cannot be concluded from our data. Were causative relationships to be established through further studies, then personalised nutritional and supplementation strategies should consider individual conditions and disease-specific factors to optimise essential metal balance and reduce CVD risk through tailored dietary adjustments and supplementation.

## 5. Conclusions

The findings detailed in this study highlight a significant association between imbalances in magnesium, copper, and zinc levels and an increased risk of MACEs and diseases of the circulatory system. Generally, low magnesium, high copper, and low zinc levels were linked to a higher risk across most conditions examined. Also notably, low magnesium appeared to have a potential protective effect for certain conditions, including acute and chronic rheumatic diseases, ischaemic diseases, and pulmonary heart diseases. Low copper levels were associated with an elevated risk of venous and lymphatic diseases, while both high and low zinc levels correlated with an increased risk of other unspecified circulatory disorders. These results provide valuable insights into the intricate relationship between trace metal homeostasis and cardiovascular health within a Scottish population. They also underscore the importance of targeted monitoring and potential interventions to mitigate CVD risk.

## Figures and Tables

**Table 1:** ICD-10 codes and corresponding disease types defined as MACE endpoints in this study, as taken from the references [17,18].

ICD-10 Code	Disease Category
I00–I02	Acute rheumatic fever
I05–I09	Chronic rheumatic heart diseases
I10–I15	Hypertensive diseases
I20–I25	Ischaemic heart diseases
I26–I28	Pulmonary heart disease and diseases of pulmonary circulation
I30–I52	Other forms of heart disease
I60–I69	Cerebrovascular diseases
I70–I78	Diseases of arteries, arterioles, and capillaries
G45.0	Vertebro-basilar artery syndrome
G45.1	Carotid artery syndrome (hemispheric)
G45.2	Multiple and bilateral precerebral artery syndromes
G45.3	Amaurosis fugax
G45.4	Transient global amnesia
G45.8	Other transient cerebral ischaemic attacks and related syndromes
G45.9	Transient cerebral ischaemic attack, unspecified
G46.0	Middle cerebral artery syndrome
G46.1	Anterior cerebral artery syndrome
G46.2	Posterior cerebral artery syndrome
G46.3	Brain stem stroke syndrome
G46.4	Cerebellar stroke syndrome

**Table 2:** Sample sizes of MACE patients and controls within the magnesium, copper, and zinc test groups. **** *p* < 0.0001, and * *p* < 0.05.

	Magnesium					
Status	High		Normal		Low	
Total number	4164		104,818		15,704	
Group	MACE patients	Control	MACE patients	Control	MACE patients	Control
Number	1223	2941	113,886	410,181	5455	10,249
Percentages	29.4%	70.6%	27.9%	72.1%	34.7%	65.3%
*p*-value	High vs. Normal; *p* < 0.0001 ****			Normal vs. Low; *p* < 0.0001 ****		
	**Copper**					
Status	High		Normal		Low	
Total number	568		3361		101	
Group	MACE patients	Control	MACE patients	Control	MACE patients	Control
Number	103	465	492	2869	10	91
Percentages	18.1%	81.9%	14.6%	85.4%	9.9%	90.1%
*p*-value	High vs. Normal; *p* = 0.0316 *			Normal vs. Low; *p* = 0.1828		
	**Zinc**					
Status	High		Normal		Low	
Total number	64		1893		404	
Group	MACE patients	Control	MACE patients	Control	MACE patients	Control
Number	6	58	164	1729	73	331
Percentages	9.4%	90.6%	8.7%	91.3%	18.1%	81.9%
*p*-value	High vs. Normal; *p* = 0.8424			Normal vs. Low; *p* < 0.0001 ****		

**Table 3:** Effects of high and low magnesium levels on disease occurrence against normal levels. The odds ratios (ORs) and 95% confidence intervals (CIs) for high or low levels relative to normal levels are presented. *** *p* < 0.001, ** *p* < 0.01, and * *p* < 0.05.

ICD-10 Codes	Diseases of the Circulatory System	OR for High Magnesium	95%CI for High Magnesium	OR for Low Magnesium	95%CI for Low Magnesium
I00–I02	Acute rheumatic fever	1.70	[0.77, 3.73]	0.27 **	[0.11, 0.68]
I05–I09	Chronic rheumatic heart diseases	1.17 ***	[1.09, 1.26]	0.82 ***	[0.78, 0.85]
I10–I15	Hypertensive diseases	0.99	[0.96, 1.02]	1.07 ***	[1.05, 1.08]
I20–I25	Ischaemic heart diseases	0.97 *	[0.93, 1.00]	0.93 ***	[0.91, 0.94]
I26–I28	Pulmonary heart disease and diseases of pulmonary circulation	0.94 **	[0.90, 0.98]	0.96 ***	[0.94, 0.98]
I30–I52	Other forms of heart disease	1.10 ***	[1.07, 1.13]	1.03 ***	[1.02, 1.05]
I60–I69	Cerebrovascular diseases	0.96 *	[0.93, 0.99]	1.07 ***	[1.05, 1.09]
I70–I79	Diseases of arteries, arterioles and capillaries	1.06 **	[1.02, 1.11]	1.10 ***	[1.08, 1.12]
I80–I89	Diseases of veins, lymphatic vessels and lymph nodes, not elsewhere classified	0.94	[0.90, 0.98]	1.22 ***	[1.19, 1.25]
I95–I99	Other unspecified disorders of the circulatory system	1.04 *	[1.00, 1.07]	1.21 ***	[1.19, 1.23]

**Table 4:** Effects of high and low copper levels on disease occurrence against normal levels. The odds ratios (ORs) and 95% confidence intervals (CIs) for high or low levels relative to normal levels are presented. *** *p* < 0.001, ** *p* < 0.01, and * *p* < 0.05.

ICD-10 Codes	Diseases of the Circulatory System	OR for High Copper	95%CI for High Copper	OR for Low Copper	95%CI for Low Copper
I00–I02	Acute rheumatic fever	No data available			
I05–I09	Chronic rheumatic heart diseases	2.26 *	[1.07, 4.76]	1.03	[0.14, 7.99]
I10–I15	Hypertensive diseases	1.78 ***	[1.40, 2.26]	0.51	[0.24, 1.10]
I20–I25	Ischaemic heart diseases	1.64 **	[1.19, 2.25]	0.26	[0.06, 1.06]
I26–I28	Pulmonary heart disease and diseases of pulmonary circulation	1.52	[0.96, 2.41]	0.58	[0.14, 2.36]
I30–I52	Other forms of heart disease	1.37	[1.07, 1.76]	1.21	[0.73, 2.00]
I60–I69	Cerebrovascular diseases	1.62 **	[1.17, 2.23]	0.28	[0.07, 1.13]
I70–I79	Diseases of arteries, arterioles and capillaries	1.48 *	[1.01, 2.16]	0.75	[0.27, 2.04]
I80–I89	Diseases of veins, lymphatic vessels and lymph nodes, not elsewhere classified	1.37	[0.98, 1.91]	2.64 ***	[1.59, 4.38]
I95–I99	Other unspecified disorders of the circulatory system	1.20	[0.90, 1.59]	1.19	[0.67, 2.11]

**Table 5:** Effects of high and low zinc levels on disease occurrence against normal levels. The odds ratios (ORs) and 95% confidence intervals (CIs) for high or low levels relative to normal levels are presented. *** *p* < 0.001, ** *p* < 0.01, and * *p* < 0.05.

ICD-10 Codes	Diseases of the Circulatory System	OR for High Zinc	95%CI for High Zinc	OR for Low Zinc	95%CI for Low Zinc
I00–I02	Acute rheumatic fever	No data available			
I05–I09	Chronic rheumatic heart diseases	1.77	[0.22, 14.05]	1.45	[0.49, 4.35]
I10–I15	Hypertensive diseases	0.66	[0.29, 1.51]	1.38 *	[1.02, 1.87]
I20–I25	Ischaemic heart diseases	1.14	[0.41, 3.17]	2.28 ***	[1.53, 3.40]
I26–I28	Pulmonary heart disease and diseases of pulmonary circulation	2.68	[0.92, 7.81]	1.77	[0.94, 3.35]
I30–I52	Other forms of heart disease	1.26	[0.61, 2.64]	2.58 ***	[1.94, 3.43]
I60–I69	Cerebrovascular diseases	0.37	[0.05, 2.73]	2.93 ***	[1.91, 4.48]
I70–I79	Diseases of arteries, arterioles and capillaries	2.23	[0.94, 5.29]	1.82 *	[1.14, 2.90]
I80–I89	Diseases of veins, lymphatic vessels and lymph nodes, not elsewhere classified	2.41	[0.93, 6.26]	3.84 ***	[2.44, 6.04]
I95–I99	Other unspecified disorders of the circulatory system	2.93 **	[1.52, 5.67]	3.00 ***	[2.11, 4.27]

## Data Availability

The original contributions presented in this study are included in the article material. Further inquiries can be directed to the corresponding authors. The original patient data used in the study are available via application to the Health Informatics Centre at the University of Dundee, UK.

## Abbreviations

The following abbreviations are used in this manuscript:

CHI	Community health index
CI	Confidence interval
CVD	Cardiovascular disease
EHRs	Electronic health records
glm	Generalised linear model
HIC	Health Informatics Centre
ICD-10	International Classification of Diseases, 10th Revision
MACEs	Major adverse cardiovascular events
MI	Myocardial infarction
NHS	National Health Service
OR	Odds ratio
PROCHI	Pseudo-CHI
SMRs	Scottish morbidity records
WHO	World Health Organisation
